# Development and application of a 40K liquid chip enriched with structural variation markers for watermelon breeding

**DOI:** 10.1093/hr/uhag221

**Published:** 2026-06-03

**Authors:** Mingxia Zhao, Rui Cheng, Yongchun Wang, Jisheng Liu, Xiaoni Li, Lifang Xue, Taohong Fang, Yudong Sun, Yun Deng, Yunbi Xu, Xingping Zhang

**Affiliations:** Crop Genetics and Breeding Platform, Peking University Institute of Advanced Agricultural Sciences, Shandong Laboratory of Advanced Agricultural Sciences in Weifang, Weifang, Shandong 261325, China; Vegetable Research and Development Center, Huaiyin Institute of Agricultural Sciences of Xuhuai Region in Jiangsu, Huai’an, Jiangsu 223001, China; Crop Genetics and Breeding Platform, Peking University Institute of Advanced Agricultural Sciences, Shandong Laboratory of Advanced Agricultural Sciences in Weifang, Weifang, Shandong 261325, China; Crops Division, Molbreeding Biotechnology Co., Ltd., Shijiazhuang, Hebei 050000, China; Crop Genetics and Breeding Platform, Peking University Institute of Advanced Agricultural Sciences, Shandong Laboratory of Advanced Agricultural Sciences in Weifang, Weifang, Shandong 261325, China; Crop Genetics and Breeding Platform, Peking University Institute of Advanced Agricultural Sciences, Shandong Laboratory of Advanced Agricultural Sciences in Weifang, Weifang, Shandong 261325, China; Crop Genetics and Breeding Platform, Peking University Institute of Advanced Agricultural Sciences, Shandong Laboratory of Advanced Agricultural Sciences in Weifang, Weifang, Shandong 261325, China; Vegetable Research and Development Center, Huaiyin Institute of Agricultural Sciences of Xuhuai Region in Jiangsu, Huai’an, Jiangsu 223001, China; Crop Genetics and Breeding Platform, Peking University Institute of Advanced Agricultural Sciences, Shandong Laboratory of Advanced Agricultural Sciences in Weifang, Weifang, Shandong 261325, China; Crop Genetics and Breeding Platform, Peking University Institute of Advanced Agricultural Sciences, Shandong Laboratory of Advanced Agricultural Sciences in Weifang, Weifang, Shandong 261325, China; Crop Genetics and Breeding Platform, Peking University Institute of Advanced Agricultural Sciences, Shandong Laboratory of Advanced Agricultural Sciences in Weifang, Weifang, Shandong 261325, China

## Abstract

Watermelon (*Citrullus lanatus*) has undergone severe loss of genetic diversity due to domestication, necessitating the exploitation of wild relatives for trait improvement. While structural variations (SVs) represent a major source of genetic diversity, high-throughput SV genotyping remains technically challenging, and existing single-nucleotide polymorphism (SNP) arrays fail to capture presence/absence variations (PAVs) that differentiate wild from cultivated germplasm. Here, we developed the ‘Watermelon 40K SNP-SV Chip,’ the first liquid-phase genotyping chip enriched with SV markers, leveraging a telomere-to-telomere super-pangenome of seven *Citrullus* species. The chip comprises 7087 SV markers (enriched for PAVs) and 11 383 SNP/InDel markers. Technical validation in 12 accessions and large-scale genotyping of 228 diverse accessions demonstrated high reproducibility (≥98.5% concordance) and robust call rates (>97%), though performance declined in deeply divergent wild species. Genome-wide association studies revealed SV-specific signals for flesh color and rind pattern that co-localized with known quantitative trait loci (QTL), including a strong candidate regulatory PAV upstream of *LCYB*. The predominance of rare variants (74.3% SVs with minor allele frequency < 0.05) limits statistical power for detecting QTL underlying polygenic domestication traits such as yield but confers exceptional utility for tracking wild introgressions in early-generation breeding populations. The 40K SNP-SV chip bridges the gap between fundamental SV research and practical breeding, enabling SV-assisted genomic selection for watermelon improvement.

## Introduction

Watermelon (*Citrullus lanatus* subsp. *vulgaris*) is one of the most economically important fruit crops worldwide. However, long-term domestication and strong artificial selection have significantly reduced its genetic diversity, leading to the loss of certain disease-resistance traits and adaptability to abiotic stresses, which, in turn, constrains the potential for genetic improvement in cultivated watermelon [1]. In contrast, wild relatives harbor abundant genetic variation, serving as key resources for broadening the genetic base of cultivated varieties and accelerating molecular breeding [2]. Currently, watermelon breeding has entered the genome-driven era, where progress largely depends on the systematic mining and efficient utilization of various genomic variations.

While single-nucleotide polymorphisms (SNPs) have been widely used in constructing genetic maps and mapping major agronomic traits [3–8], structural variations (SVs), including deletions, insertions, duplications, and inversions, represent other important forms of genetic variation, often associated with stronger phenotypic effects and adaptive significance, holding great potential for crop improvement [9]. Therefore, the systematic identification and evaluation of SVs in watermelon are of considerable value for breaking through existing breeding bottlenecks and mining superior alleles from wild genetic resources.

The high-throughput genotyping of SVs, however, still faces significant technical bottlenecks. Short-read resequencing, the mainstream technology for population genetic analysis, has systematic limitations in SV detection. Its assembly, calling, and genotyping procedures are error-prone, not only leading to a high false-negative rate but also introducing length-dependent ascertainment bias, which severely compromises the reliability of subsequent association analyses [10]. Consequently, many functionally important SVs remain ‘hidden’. In this context, graph-based pangenomes provide a powerful framework that greatly enhances the detection of diverse SVs, which constitute a substantial fraction of genomic diversity and have been established as significant determinants of phenotypic variation [7, 11–13]. Building on this advance, our previous study constructed the first telomere-to-telomere (T2T) graph-based super-pangenome for the genus *Citrullus* [2]. This work systematically identified >460 000 SVs and, using structural variant genome‑wide association study (SV‑GWAS), uncovered key genes that were lost or gained during domestication, highlighting the unique value of SVs in improving the resolution of complex trait dissection. Nevertheless, translating these foundational discoveries into practical breeding tools remains challenging. Currently, conventional SNP arrays cannot effectively capture SV information, whereas whole-genome resequencing–based SV genotyping is costly and analytically complex [14], limiting its scalability for breeding populations. Therefore, developing a technical platform capable of efficient, accurate, and cost-effective large-scale SV genotyping has become a critical gap bridging fundamental SV research and molecular breeding practices in watermelon.

Recently, Genotyping by Target Sequencing (GBTS) has emerged as a viable solution to the aforementioned challenges. By utilizing probe-based capture and high-throughput sequencing of specific genomic loci, GBTS combines the high stability of solid-phase chips with the flexibility and cost-effectiveness of genotyping-by-sequencing [15]. This technology has been successfully applied in various crops, such as maize [16], wheat [17], cotton [18], pepper [19], and melon [20]. Notably, Duan *et al*. [21] recently developed a SNP-based liquid chip for watermelon using resequencing data from 76 cultivated accessions. While this represents a significant advance for assessing within-cultivar diversity, such SNP-centric platforms inherently overlook large-effect SVs, especially presence/absence variations (PAVs) and gene copy number variations that distinguish wild species from cultivated forms and frequently underlie key agronomic traits.

To fill this critical gap, here we leverage the first T2T super-pangenome of the *Citrullus* genus [2] to develop ‘Watermelon 40 K SNP-SV Chip’, the first high-throughput liquid-phase genotyping chip specifically dedicated to SVs in watermelon. The 40K platform accommodates up to 90 000 probes. We synthesized 86 380 probes (averaging ~4.7 per locus) for the multiplexed capture of 18 470 variant loci, ensuring robust detection of diverse SV architectures. Unlike previous SNP arrays, the 40K SNP-SV Chip directly captures the ‘presence–absence’ dimension of genomic diversity, enabling efficient tracking of trait introgression and regulatory SVs undetectable by single-nucleotide assays. The chip was designed from a high-quality SV catalogue derived from 28 accessions spanning all seven *Citrullus* species, comprising 7087 SV and 11 383 SNP/InDel markers. Its performance was evaluated by genotyping both representative and diverse germplasm accessions. The chip has been used in genome-wide association studies (GWAS) on rind pattern and flesh color identified multiple SV loci surpassing the Bonferroni-corrected threshold, co-localizing with previously reported QTL.

## Results

### Design and genomic distribution of the Watermelon 40K chip markers

The Watermelon 40K SNP-SV Chip was developed from a super-pangenome resource encompassing 28 accessions across seven *Citrullus* species (Fig. 1A). Building upon the super-pangenome resource that identified ~460 000 high-confidence SVs [2], we expanded the candidate pool to 6.9 million SVs and 24.4 million SNPs for probe design by including lower frequency variants from all seven species, we implemented a stringent filtration pipeline assessing breakpoint clarity, flanking sequence uniqueness, guanine-cytosine (GC) content (30%–70%), probe specificity, and optimal probe length (110 bp). This process yielded 18 470 high-quality loci, comprising 11 383 SNP/InDel markers and 7087 SV markers.

**Figure 1:**
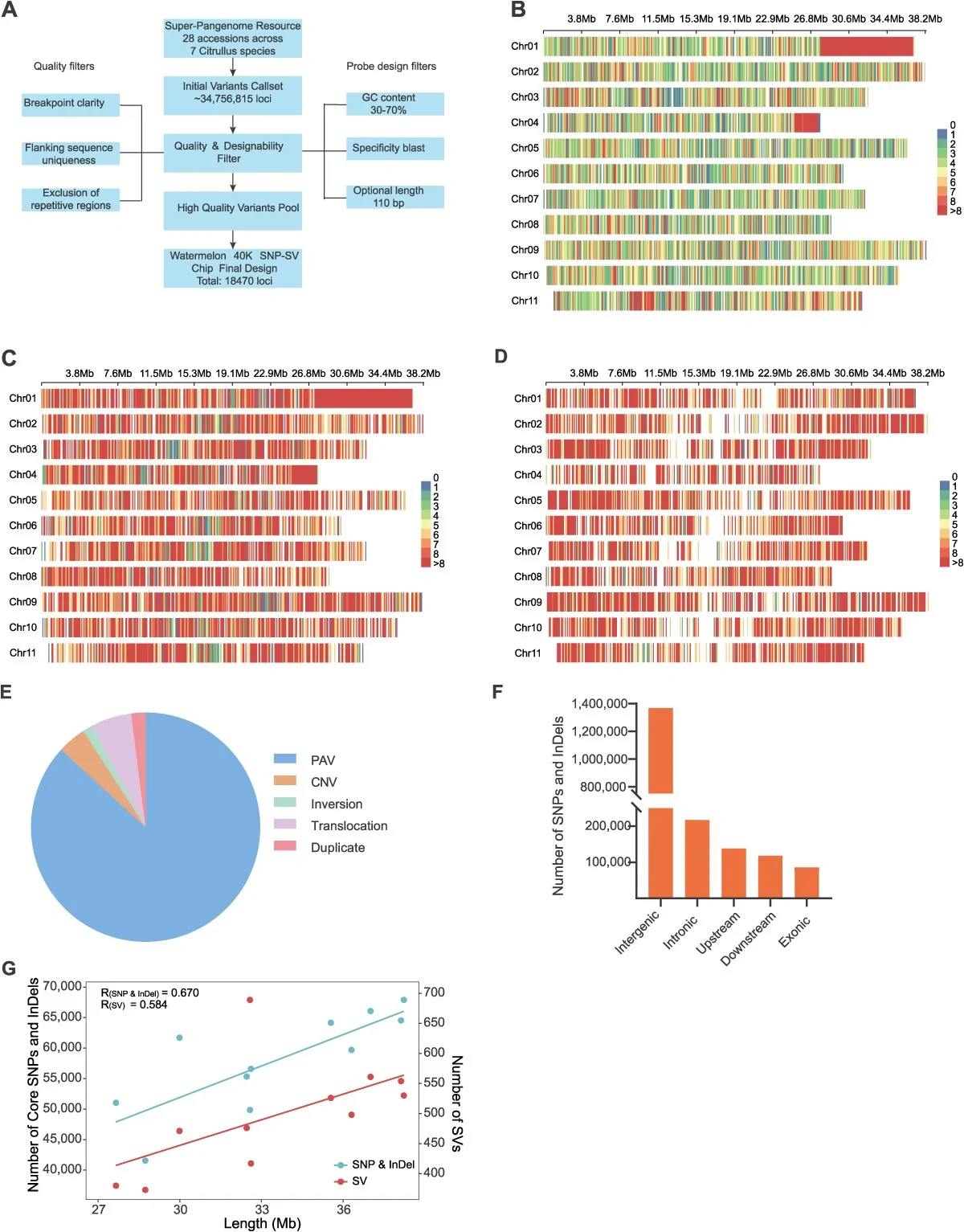
Design and genomic features of the Watermelon 40K liquid chip enriched with structural variation (SV) markers. (A) Workflow for chip development. (B–D) Chromosomal distribution of markers: (B) all 18 470 loci, (C) 11 383 SNP and InDel markers, and (D) 7087 SV markers. (E) Composition of SV types in the chip. PAV, presence/absence variation; CNV, copy number variation. (F) Distribution of SNPs and InDels markers on gene structure. (G) Correlation between chromosome length and number of variants.

The final chip composition reflects severe differential attrition across variant classes during probe design and spacing-based filtering (Table 1). From an initial discovery pool of 24 420 874 SNPs and 6 851 083 SVs (including 6 777 958 InDels, 66 501 PAVs, and 7624 complex SVs) identified across the super-pangenome, only 18 470 loci (0.05%) ultimately met the stringent criteria for probe synthesis. This funnel effect operates at two distinct stages: probe design feasibility and genomic spacing filtering. Probe design feasibility varied dramatically by variant architecture: PAVs achieved the highest success rate (32.6%), benefiting from unique flanking sequences, while InDels showed intermediate success (25.2%) but extreme density-dependent attrition. SNPs exhibited the lowest per-locus success (14.6%), reflecting stringent specificity filters, yet their vast initial pool (24.4 M) ensured adequate final marker numbers. These metrics establish that SV marker development requires 10 ~ 100-fold larger initial candidate pools than SNP markers, with InDels presenting the most severe bottleneck. In total, 86 380 probes were synthesized, utilizing 96% of the 90 000-probe platform capacity (Table S1).

**Table 1:** Marker development efficiency and statistics for the Watermelon 40K SNP-SV Chip

**Variant class**	**Initial candidates**	**Probes assessed**	**Design success (%)**	**Spacing filter**	**Final in array**	**Overall yield (%)**	**No. of probes**	**Avg probes/locus**
SNP	24 420 874	34 514	14.6	200 bp	5114	0.02	8296	1.6
InDel	6 777 958	1 708 303	25.2	1900 bp	6269	0.09	37 614	6
PAV	66 501	21 676	32.6	Dual-flank	6154	9.3	36 990	6
CNV	1948	619	31.8	Dual-flank	285	14.6	1140	4
Inversion	751	103	13.7	None	103	13.7	297	2.9
Translocation	2184	402	18.4	None	402	18.4	1608	4
Duplicate	1741	143	8.2	None	143	8.2	435	3
Total	34 756 815	1 765 676			18 470	0.05	86 380	4.7

Chromosomal distribution analysis revealed comprehensive coverage across all 11 watermelon chromosomes without significant gaps (Fig. 1B). However, distinct distribution patterns emerged between marker classes. While SNP/InDel markers exhibited relatively even dispersal across chromosomal arms (Fig. 1C), SV markers displayed a bipolar distribution with significant enrichment at chromosome arms and conspicuous gaps in pericentromeric regions (Fig. 1D). These gaps correspond to centromeric satellite repeats where probe design is challenging due to mapping ambiguity, resulting in SV markers predominantly reside within gene-rich euchromatic regions.

Structural variation composition analysis showed that PAVs constituted the majority, followed by copy number variations (CNVs), inversions, translocations, and duplications (Fig. 1E). Regarding gene structure, SNPs and InDels were predominantly located in intergenic regions, with progressively fewer variants in intronic, upstream, downstream, and exonic sequences (Fig. 1F). As expected, marker counts correlated positively with chromosome length (*R* = 0.670 for SNPs/InDels; *R* = 0.584 for SVs) (Fig. 1G).

This chip represents the first high-throughput liquid-phase genotyping platform specifically dedicated to capturing SVs in watermelon. The enrichment of PAV-type SVs within genic regions underscores the chip’s functional relevance for detecting trait-associated SVs that are undetectable by conventional SNP arrays.

### Technical validation and performance evaluation of the Watermelon 40K SNP-SV Chip

To evaluate the robustness of the 40 K SNP-SV Chip across different genetic backgrounds, we genotyped 12 representative accessions spanning the *Citrullus* genus, including seven cultivated *C. lanatus* accessions and five wild relatives. However, the most genetically divergent species, the dioecious *C. naudiananus*, yielded an unacceptably low call rate of 47.15%, falling below the 50% quality threshold, and was consequently excluded from downstream analysis. The remaining 11 accessions revealed a striking correlation between genetic distance from cultivated germplasm and probe performance. Cultivated watermelons consistently exhibited high call rates (>98%), whereas wild species showed a gradient of performance. *Citrullus mucosospermus* (PI 595203), a wild relative closely related to cultivated watermelon, achieved 98.0%, concordant with cultivar levels; *C. amarus* (PI 482276) reached 86.04%; *C. rehmii* and *C. ecirrhosus* maintained 79.51% and 78.47%, respectively; while the most distant relative, *C. colocynthis* (PI 537300), yielded 77.19% (Table S2).

Principal component analysis (PCA) of the qualified genotyping data clearly distinguished cultivated from wild germplasm along PC1 (34.5%) and PC2 (25.8%), with cultivated varieties forming a tight cluster distinct from the dispersed wild accessions (Fig. 2A). Notably, call rate performance correlated strongly with this genetic stratification (Fig. 2B). This gradient reflects the ascertainment bias inherent in the chip design, as probes were developed primarily from cultivated genomes. As a result, accessions harboring highly divergent or wild-specific haplotypes exhibited elevated missing data rates due to inefficient probe hybridization. The failure to genotype *C. naudiananus*, a species representing the deepest divergence in the genus, highlights the current limitation for extremely divergent germplasm. Nevertheless, the >75% call rates achieved across the remaining wild species indicate acceptable performance for preliminary introgression screening, while the exceptional performance of *C. mucosospermus* suggests that the chip is particularly well suited for capturing variation within the primary gene pool of watermelon.

**Figure 2:**
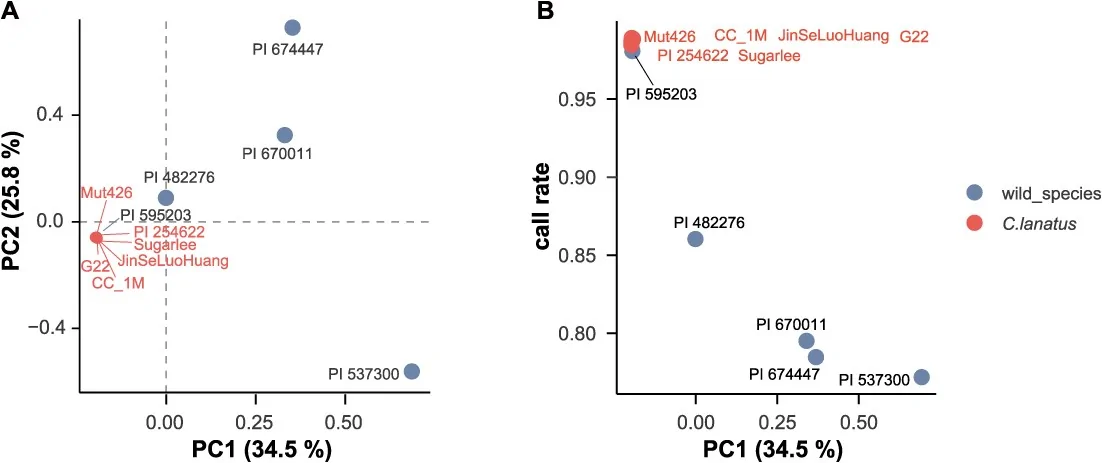
Technical validation of the 40K SNP-SV Chip using a 12-accession diversity panel. (A) Principal component analysis (PCA) of 11 accessions covering six *Citrullus* species.Wild species and *C. lanatus* cultivars are distinguished as indicated in the figure legend. (B) Correlation between call rate and genetic distance (represented by PC1 coordinate).

Based on pilot test results revealing low call rates (<80%) in highly divergent wild species such as *C. colocynthis* and *C. amarus*, the large-scale validation panel was strategically restricted to 228 accessions comprising two germplasm groups: *C. mucosospermus* (egusi type, *n* = 7) and *C. lanatus* (*n* = 221), the latter encompassing cultivated varieties, landraces, and wild accessions primarily from Africa, West Asia, and Asia. This selection ensured robust genotyping performance while capturing the primary domestication diversity within the genus (Fig. 3A). Among these, 220 accessions exhibited high call rates (>90% Fig. S1), while 8 accessions (primarily wild species with high divergence) showed lower call rates (<85%) but were retained in the dataset to maximize diversity representation.

**Figure 3:**
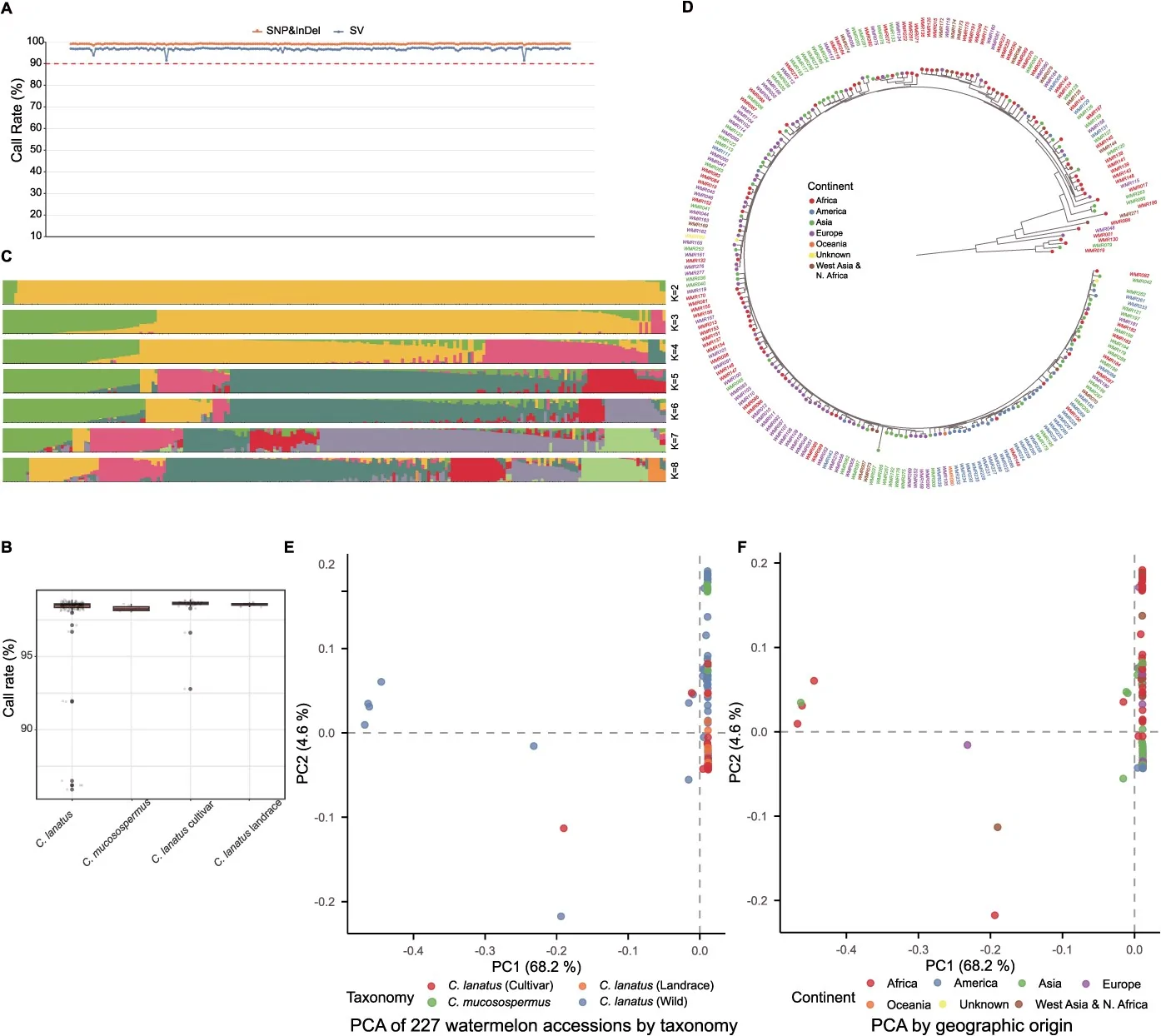
Large-scale genotyping performance in a 228-accession natural population. (A) Comparison of call rates between SNP & InDel and SV markers across all samples. (B) Call rate distribution across different taxonomic groups. (C) ADMIXTURE analysis of 228 watermelon accessions for *K* = 2–8. (D) Neighbor-joining phylogenetic tree of 228 accessions with geographic origin indicated (see legend). (E) PCA based on taxonomic classification. (F) PCA based on geographic origin.

#### Technical validation of the 40K SNP-SV Chip revealed high genotyping quality across all samples

The call rates were consistently high (mean > 95%) with uniform performance across different taxonomic groups, including wild *C. lanatus*, *C. mucosospermus*, landraces, and cultivars, indicating no systematic bias against divergent germplasm (Fig. 3B). Furthermore, sequencing depth positively correlated with call rate (*r* = 0.585, *P* < .001), confirming that the genotyping quality was depth-dependent and technically robust (Fig. S2).

Population structure analysis using ADMIXTURE (*K* = 1–8) and PCA revealed a highly coupled geographic–taxonomic dual structure concordant with the African single-origin model. When *K* = 2, the population were divided into two distinct clusters: the African–West Asian wild reservoir (including both *C. mucosospermus* and wild *C. lanatus*) and the Asia–America-cultivated germplasm, reflecting the primary divergence between ancestral and derived gene pools (Fig. 3C). As *K* increased to 3, geographic differentiation emerged with the separation of African wild from Asian-cultivated clusters, while landraces displayed substantial admixture between these gene pools. Interestingly, from *K* = 4, *C. mucosospermus* began to differentiate from the West African wild *C. lanatus*, displaying distinct but nonindependent genetic components embedded within the African clade (Pop2: 71.4%, Pop3: 28.6%), whereas a sophisticated genetic structure was observed with the emergence of a rare, highly differentiated Pop4 lineage (2.6% of accessions, mean purity 83.5%) (Fig. 3C). Phylogenetic analysis further supported the ADMIXTURE results, revealing a clear evolutionary separation between the basal African–West Asian wild clade and the derived Asia–America-cultivated clade. *Citrullus mucosospermus* accessions were embedded within the West African branch, forming a monophyletic group distinct from but phylogenetically proximate to wild *C. lanatus*, rather than constituting a distant outgroup (Fig. 3D).

Synthesizing the phylogenetic tree, geographic PCA, and taxonomic PCA data unveiled distinct evolutionary patterns. PC1 (68.2% variance) captured both species divergence and geographic isolation, separating the African–West Asian wild reservoir from the Asia–America-cultivated germplasm. PC2 (4.6% variance) resolved the domestication gradient within *C. lanatus*, with landraces forming a transitional bridge between wild (basal/outgroup position in phylogeny) and cultivar clusters (Fig. 3E and F). Statistical validation revealed minimal genetic differentiation between *C. mucosospermus* and African *C. lanatus*, supporting genetic continuity consistent with their status as congeneric species within the primary gene pool, likely facilitated by ongoing or historical gene flow in West Africa. Notably, low-frequency wild ancestry signals were detected within cultivated clusters, indicating minimal postdomestication introgression that did not substantially alter the cultivated genomic background.

#### Robustness of structural variation–inferred population structure and complementarity of multimarker principal component analysis

To evaluate whether the population structure inferred from the 40K SNP-SV Chip is robust across marker classes and to address potential concerns regarding SV-specific ascertainment bias, we compared PCA results derived from three complementary marker sets: SV, SNP/InDel, and chip markers (total marker set). The PC1 from SV analysis explained 34.3% of the genetic variance and exhibited near-perfect correlation with that from the Total marker set (Pearson’s *r* = 0.990; Fig. S3). This near-identical stratification of cultivated versus wild germplasm demonstrates that SVs, despite their lower marker density, distinct genomic distribution (enriched in gene-rich euchromatic arms; Fig. 1D), and higher design attrition rate, faithfully capture the primary domestication gradient without systematic bias. The substantially higher variance explained by PC1 in the Total analysis (68.2%) therefore reflects the additive contribution of both variant classes rather than a qualitatively distinct population structure.

Intriguingly, SV PCA revealed enhanced resolution of wild progenitor relationships. The PC2 explained 11.4% of variance in SV analysis, more than double that in Total analysis (4.6%), and delineated clearer geographic substructure among African wild accessions (Fig. S3). This enhanced resolution is consistent with the molecular properties of SVs: lineage-specific PAVs are less prone to homoplasy than SNPs and preserve deeper phylogenetic signals, making them particularly informative for resolving relationships among closely related wild lineages. Conversely, SNP/InDel PCA exhibited intermediate PC2 resolution (7.8% variance explained) and clustered wild accessions more tightly, suggesting that the high density and rapid mutation rate of short variants partially obscure ancient population splits.

Collectively, these comparisons confirm that the 40K SNP-SV Chip provides unbiased, robust population genetic inference and offers unique evolutionary insights into wild germplasm differentiation that are partially masked in SNP-dominated datasets. This complementary information content, SVs for deep lineage tracing, SNPs for high-resolution admixture detection, underscores the value of integrating multiple variant classes in population genomic studies of watermelon and other crops with complex domestication histories.

Leveraging this enhanced phylogenetic resolution, we re-examined the evolutionary placement of *C. mucosospermus* and the overall germplasm structure. The delayed differentiation of *C. mucosospermus* (until *K* = 4) and its admixture pattern (primarily Pop2/3 components without Pop4 contribution) position it as a specialized West African lineage with substantial genetic continuity to wild *C. lanatus*, rather than a distant outgroup. This pattern likely reflects continuous or historical gene flow between *C. mucosospermus* and *C. lanatus* in West Africa, coupled with relatively low genetic divergence compared to intercontinental differentiation. In addition, the emergence of Pop4 at *K* = 4 suggests the presence of a highly differentiated ancestral lineage possibly representing isolated genetic diversity or extreme drift effects, warranting further investigation as a potential new genetic resource.

These results collectively define three operationally distinct germplasm pools: (i) the basal Africa–West Asia wild reservoir (Pop1 and admixed African accessions, representing the ancestral outgroup harboring stress-resistance and bitter-compound genes); (ii) the specialized *C. mucosospermus* lineage (embedded within West African Pop2/3, genetically proximate to wild *C. lanatus* but characterized by unique seed mucilage traits, representing a distinct evolutionary unit within the primary gene pool); and (iii) the derived Asia–America cultivated pool (landraces and elite cultivars with minimal wild introgression, enriched for domestication traits such as sweetness and red flesh). Notably, the Pop4 lineage represents a rare, potentially ancestral component distinct from all three pools. The 40K SNP-SV Chip accurately resolves these phylogenetic relationships, distinguishing ancestral wild diversity from the divergent *C. mucosospermus* and the derived cultivars. This capability establishes its utility for targeted introgression, whether mining stress-resistance alleles from the wild reservoir or introgressing seed-quality traits from *C. mucosospermus*, while enabling structure-aware genetic analyses to minimize population stratification artifacts in downstream GWAS.

### Population genomic characteristics: allele frequency spectrum and linkage disequilibrium

To characterize the genetic properties of the 40K SNP-SV Chip markers for association mapping, we analyzed the allele frequency spectrum and linkage disequilibrium (LD) patterns using the 228-accession validation panel. The minor allele frequency (MAF) distributions in the 228-accession validation panel revealed an extremely left-skewed site frequency spectrum for both marker classes (Fig. 4A). Notably, 67.0% of SNPs/InDels and 74.3% of SVs were concentrated within the rare variant range (MAF < 0.05), with the 0–0.05 bin representing the majority loci (Fig. S4). This distribution reflects the ascertainment bias inherent to the chip design, which prioritized capturing low-frequency diversity from the pangenome discovery panel.

**Figure 4:**
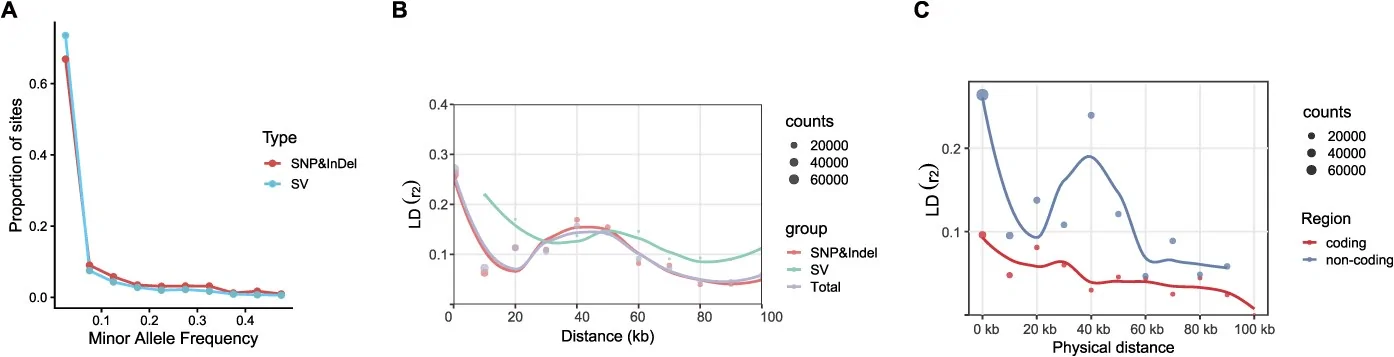
Allele frequency spectrum and linkage disequilibrium patterns in the 228-accession validation panel. (A) MAF distribution showing left-skewed spectra for both SNP/InDel and SV markers (see legend for marker type assignment), with 67.0% and 74.3% rare variants (MAF < 0.05), respectively. (B) Genome-wide LD decay curves plotted as mean *r*^2^ against physical distance for three marker categories: Total (chip markers), SNP & InDel, and SV. (C) Comparison of LD decay between coding and noncoding regions based on SNP & InDel markers.

LD decay analysis revealed distinct decay dynamics between marker classes (Fig. 4B). SNP/InDel markers exhibited rapid LD decay, dropping to *r*^2^ ≈ 0.1 within ~10–20 kb, consistent with high recombination rates and dense marker coverage across the genome. In striking contrast, SV markers showed markedly slower decay, maintaining *r*^2^ > 0.1 throughout the 10–70 kb interval, likely reflecting recombination suppression within large SVs (inversions, duplications) or their tendency to cluster in low-recombination genomic regions. The ‘Total’ marker set largely tracked the SNP/InDel curve, nearly overlapping across all distances, indicating that the overall LD landscape is dominated by the abundant SNP/InDel component. Notably, both the SNP/InDel and Total curves exhibited a secondary elevation around 25 kb (*r*^2^ ≈ 0.15) before declining again by 60 kb, potentially reflecting chromosome-scale structural features such as pericentromeric regions or segregating inversions that transiently elevate local LD. Analysis of LD decay by genomic context (Fig. 4C) further revealed that coding regions exhibited faster initial LD decay compared to noncoding regions, consistent with stronger purifying selection and higher effective recombination in gene-dense areas. These LD patterns, rapid decay for SNPs facilitating fine-mapping, and extended LD for SVs enabling long-range haplotype tracking, complement the chip’s hybrid design, optimizing it for both high-resolution association studies and detection of large-effect SVs.

### Genome-wide association studies for key fruit traits

To validate the 40K SNP-SV Chip’s efficacy for gene discovery and marker–trait association, we conducted genome-wide association studies (GWASs) on 14 fruit-related traits using a single-marker mixed linear model (EMMAX) that accounted for population structure and cryptic relatedness. Each trait was interrogated using three distinct datasets: (i) the chip loci marker set (Total, *n* = 6701 loci), (ii) SNPs and InDels (*n* = 5681), and (iii) SV haplotype blocks (*n* = 84 545), with Bonferroni-corrected significance thresholds of 5.13, 5.06, and 6.23 (−log₁₀p), respectively. For visualization, we adopted a uniform threshold of –log₁₀p = 5.0 in the Manhattan plots to allow direct comparison across datasets.

#### Genome-wide association study for flesh color

Manhattan plots for flesh color (ranging from white to red) revealed a major association signal on chromosome 4 exceeding the genome-wide significance threshold across all three datasets (Fig. 5). Using the chip markers (total marker set), the lead SNP was identified at Chr04:16353520 (−log₁₀p = 6.81, *P* = 1.55 × 10^−7^), located ~360 kb downstream of the LCYB (lycopene beta-cyclase) coding sequence, co-localizing with the known major QTL for flesh color (Guo et al., 2019; [22]). Notably, the SV haplotype blocks analysis detected a vastly stronger and more extensive association signal, with the lead marker at Chr04:15779872 (−log₁₀p = 20.76, *P* = 1.75 × 10^−21^, ~180 kb upstream of LCYB) and 11 additional significant loci spanning the interval Chr4:15779872–16235406 (Table S4).

**Figure 5:**
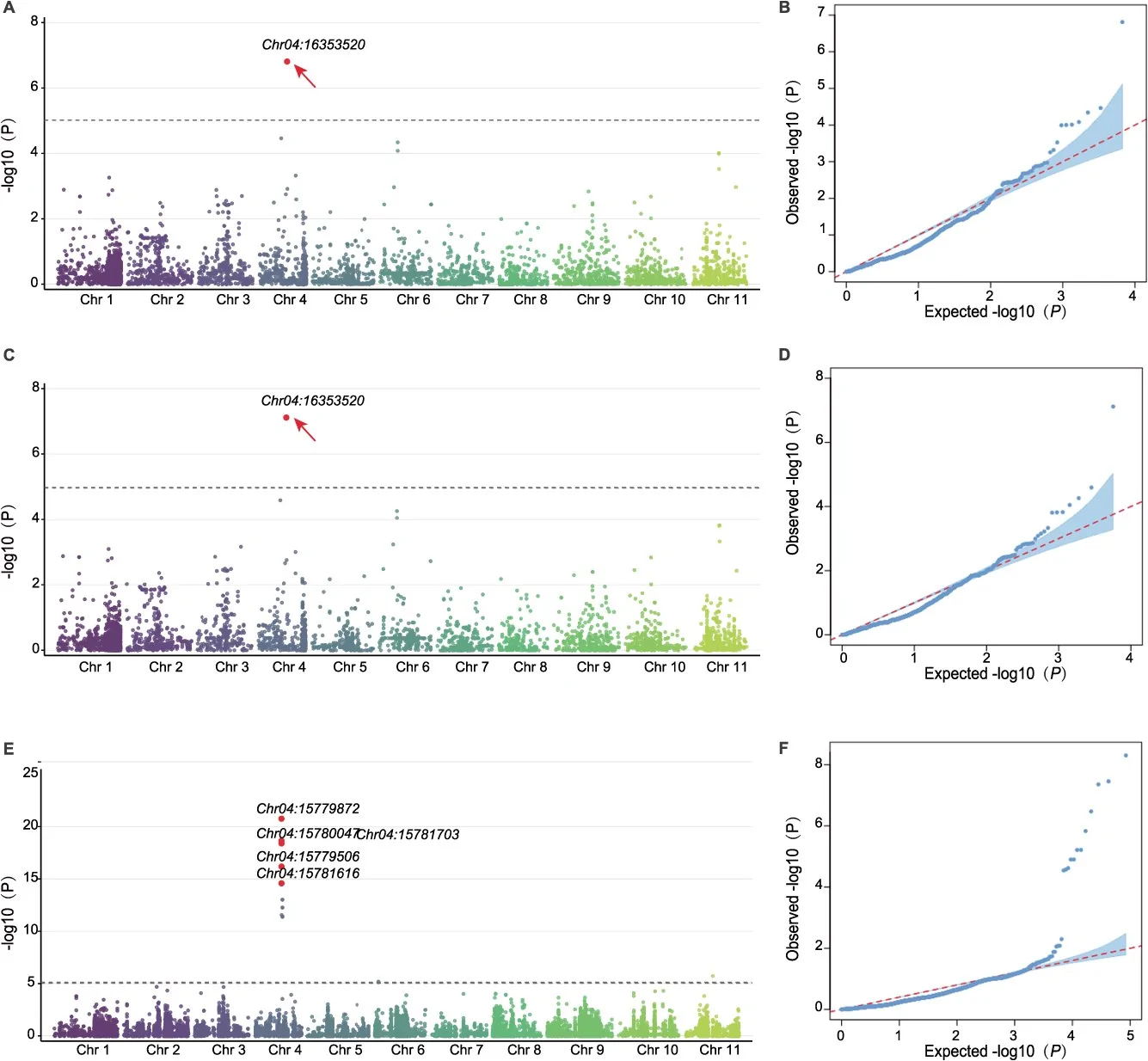
Genome-wide association mapping for flesh color. Manhattan plots (A, C, and E) and quantile–quantile (QQ) plots (B, D, F) for flesh color using three datasets: Total variants (A and B), SNPs and InDels (C and D), and SVs (E and F). The horizontal dashed lines in each Manhattan plot indicate the Bonferroni-corrected significance threshold (−log_₁₀_*P* = 5.0). The arrowhead indicates the lead signal co-localizing with the LYCB locus on chromosome 4.

In contrast to the single downstream hit captured by the SNP/InDel-based dataset, the SV haplotype blocks analysis revealed a broad association block encompassing two distinct clusters: a high-confidence upstream cluster (~15779506–15781703) and a secondary downstream cluster (~16230775–16235406). This superior resolution stems from the presence of candidate causal SVs within the SV dataset, specifically a PAV at Chr04:15872762 proximal to the upstream peak, and in the downstream region, an inversion spanning Chr04:16228037–16235627 together with a PAV at Chr04:16222617 (Table S5). These SVs are statistically associated with the locus and represent strong candidates that may influence *LCYB* expression, warranting functional validation, whereas the SNP-based association merely tags the downstream haplotype block. We note that GWAS alone cannot establish causality, and experimental validation (e.g. promoter activity assays, CRISPR/Cas9 editing) is required to confirm the regulatory role of these SVs. The failure of the SNP/InDel dataset to detect the upstream signal, despite its extreme significance in the SV analysis, highlights the critical value of SV-targeted genotyping for capturing regulatory variation that may be undetectable by conventional SNP arrays.

#### Genome-wide association study for rind pattern

For rind pattern (ordinal scores 1–6), the chip markers (total marker set) identified five significant associations clustered on chromosome 6 within the interval Chr06:28717811–29239162 (−log₁₀p ranging from 6.04 to 7.60; Fig. 6A and B). This region harbors a WDR (WD40-repeat) gene (ClG42_06g018100, Chr06:28660490–28662283), consistent with previous QTL mapping studies for rind pattern (Guo et al., 2019). Notably, this interval contains 17 PAVs, suggesting complex structural rearrangements underlying this major-effect locus. The SNP&InDel dataset detected only a single significant InDel at Chr06:28829700 (G/GA, −log₁₀p = 7.90), located within the same WDR gene cluster but failing to capture the full haplotypic diversity. In contrast, the SV haplotype blocks analysis not only confirmed the Chr6 associations (Table S4) but also revealed additional significant signals on chromosomes 1, 8, 9, and 10 (Fig. 6E and F), suggesting a polygenic architecture for rind patterning.

**Figure 6:**
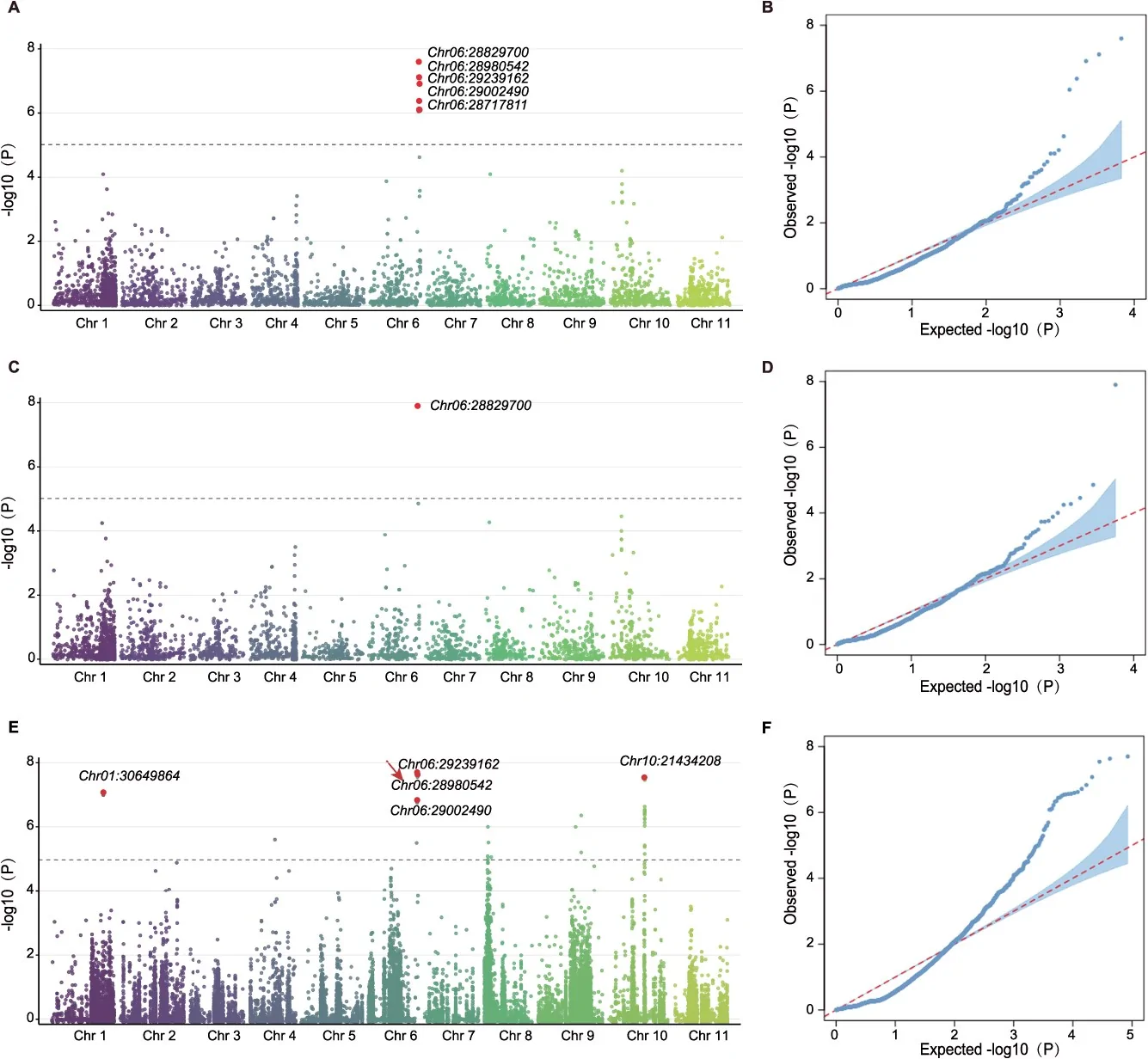
Genome-wide association mapping for rind pattern. Manhattan plots (A, C, and E) and QQ plots (B, D, and F) for rind stripe using Total variants (A and B), SNPs and InDels (C and D), and SVs (E and F). Significant associations on chromosomes 4 and 6 correspond to the previously reported St locus and a major rind-pattern QTL, respectively. The horizontal dashed line in each Manhattan plot indicates the Bonferroni-corrected significance threshold (−log₁₀*P* = 5.0). The arrowhead denote lead SV signals.

Particularly striking was the discovery of a dense cluster of 14 significant SV markers on chromosome 10 within the interval Chr10:21427369–21434492 (−log₁₀p > 6.1). This region contains ClG42_10g0099200, encoding an *S*-adenosyl-*L*-methionine-dependent methyltransferase, suggesting a potential role for epigenetic regulation (DNA methylation) in rind striping determination. Additionally, significant SV markers on chromosome 9 (Chr09:22168647 and Chr09:25488692) mapped within large cryptic SVs, a translocation (Chr09:22999917–23006866) and an inversion (Chr09:25012302–31324931) (Table S5), respectively, representing structural haplotypes completely undetectable by SNP-based assays.

These results demonstrate the superiority of SV-targeted genotyping for dissecting complex trait architecture: while SNP/InDel markers tag the primary WDR locus on Chr6, the SV analysis resolves the underlying structural complexity (56 PAVs) and discovers novel regulatory loci (methyltransferase on Chr10, cryptic SVs on Chr9), providing candidate markers that may be useful for marker-assisted selection of rind pattern traits, pending functional validation.

#### Genome-wide association study for quantitative traits

In contrast to the qualitative traits, comprehensive GWAS scans for all quantitative traits, including fruit weight, dimensions (width, length, index), pedicel length, and soluble solids content (SSC) observed in both Xiashan and Wuwei locations, failed to identify any significant associations above the genome-wide significance threshold. No significant associations were detected for fruit weight, dimensions, or SSC, consistent with the expected polygenic architecture of these domestication traits.

Collectively, these GWAS results validate that the 40K SNP-SV Chip effectively captures major-effect loci for qualitative traits, including the successful replication of known genes (LCYB) and the discovery of novel SV-based associations (methyltransferase locus for rind pattern), while highlighting limitations for detecting small-effect QTL in this particular population context.

## Discussion

### The Watermelon 40K SNP-SV Chip: a platform for capturing structural variation within the primary gene pool

The development of the Watermelon 40K chip enriched by SV markers represents a strategic evolution in crop genotyping platforms, specifically designed to bridge the resolution gap between conventional SNP arrays and whole-genome sequencing. Unlike existing SNP-centric tools that systematically miss large-effect regulatory variants, the 40K chip integrates 7087 SV markers, enriched for PAVs within gene-rich euchromatic regions, alongside 11 383 core SNP/InDel markers. The hybrid architecture, which employs multiplexed probe design (averaging ~4.7 probes per locus, including flanking and spanning probes for SVs) to ensure robust capture efficiency, is not intended to replace SNP-based genotyping but rather to complement it by capturing structural haplotypes that underlie phenotypic diversity yet remain undetectable by single-nucleotide assays.

The bipolar chromosomal distribution of SV markers that are concentrated in recombination-active arms while sparing centromeric repeats, reflects a biologically rational design choice rather than a technical shortcoming. By targeting regions where functional genes reside, the chip maximizes the probability of detecting trait-associated SVs, as evidenced by the GWAS results for flesh color and rind pattern. The predominance of PAV-type SVs (Fig. 1E) aligns with pangenome studies in other crops [12, 23, 24], suggesting that gene presence/absence polymorphisms constitute a substantial reservoir of functional diversity that SNP markers alone cannot tag.

However, the performance gradient observed across the *Citrullus* genus (Table 1) underscores a fundamental constraint inherent to array-based technologies: ascertainment bias. The progressive decline in call rates from cultivated materials (>98%) to distant wild relatives (*C. colocynthis*: 77%; *C. naudiananus*: exclusion due to <50% call rate) directly reflects the origin of probe sequences from the cultivated reference genome and the super-pangenome discovery panel. Rather than viewing this as a design flaw, we posit that this limitation operationally defines the chip’s optimal utility. Our breeding showed the hybrids derived from *C. naudiananus* are essentially sterile indicating the uniqueness of this dioecious species. The high performance in *C. mucosospermus* (98%), a wild relative crucial for seed quality improvement, coupled with moderate but suboptimal rates (>75%) in secondary gene pool species, validates the 40K as a tailored tool for the primary gene pool and early-generation introgression lines, while cautioning against its direct use for high-resolution genotyping in more distant wild relatives. Consequently, we explicitly define the chip’s operational boundary: it is optimized for the primary gene pool (*C. lanatus* cultivars, landraces, and *C. mucosospermus*) where call rates consistently exceed 95%; direct application in secondary/tertiary gene pool species (e.g. *C. colocynthis*) is not recommended for quantitative genetic studies or marker-assisted selection and should be limited to low-resolution screening (e.g. presence/absence of specific SVs) or used alongside whole-genome resequencing. This scope mirrors the deployment path of first-generation SNP arrays in maize and wheat, where initial designs prioritized elite germplasm compatibility before iterative expansion [25].

The cost-effectiveness of the liquid-phase platform (~$20/sample) positions it as a pragmatic alternative to whole-genome resequencing for routine breeding applications. While resequencing offers unbiased discovery across the entire genus, the 40K SNP-SV Chip provides sufficient resolution to capture the majority of agronomically relevant SVs within the domesticated gene pool, as demonstrated by its ability to resolve the three operational germplasm pools (basal African wild reservoir, specialized *C. mucosospermus* lineage, and derived cultivated pool) and detect regulatory SVs affecting key traits (e.g. the PAV upstream of LCYB for flesh color and the complex PAV cluster at the WDR locus for rind pattern) that SNP-based GWASs failed to reveal.

Complementary positioning of the 40K SNP-SV Chip relative to existing genotyping platforms. While Duan *et al*. [21] recently reported a valuable 40K SNP liquid chip for watermelon based on resequencing of 76 cultivated accessions, their design inherently focuses on single-nucleotide diversity within the cultivated gene pool. In contrast, the present 40K chip enriched with SV markers exploits the super-pangenome of seven *Citrullus* species, capturing PAVs and large indels that constitute the primary genomic divergence between wild relatives and cultivated forms. This distinction is biologically significant: SNP chips tag haplotype blocks through linkage disequilibrium, whereas SV markers often represent the causal variants underlying phenotypic changes (e.g. gene deletions, regulatory inversions). For instance, the PAV ~100 kb upstream of LCYB associated with flesh color in this study would be completely undetectable by SNP-centric arrays. Thus, the 40K SNP-SV Chip developed in this study does not duplicate but rather complements existing SNP chips, it fills the ‘SV gap’ in the breeder’s toolkit, enabling targeted introgression of wild alleles that SNP markers cannot effectively track due to ascertainment bias against highly divergent sequences.

### Technical constraints in single-variant array design: efficiency, bottlenecks, and lessons learned

The severe attrition funnel revealed in our development pipeline, culminating in only 0.05% of initial candidates reaching the final chip, reflects fundamental biological and technical constraints inherent to SV-targeted genotyping. While SNP arrays routinely achieve 10%–30% conversion rates from discovery to final markers, our data establish that SV development demands 10 ~ 100-fold larger initial candidate pools depending on the variant class targeted, ranging from ~2-fold for PAVs to ~50-fold for InDels and up to 100-fold for duplications, with efficiency varying dramatically by variant architecture.

The attrition operates distinctly at the probe design and genomic spacing stages. Probe design success (Table 1) is primarily constrained by sequence context: PAVs achieved the highest feasibility (32.6%) because their breakpoints often reside in unique, nonrepetitive flanking regions where 110-bp probes maintain specificity [9]. Conversely, SNPs exhibited the lowest per-locus success (14.6%), not due to technical difficulty per se but because the massive initial pool (24.4 M) permitted stringent specificity filters (BLAST ≤5, unique mapping) that eliminated ambiguous loci, a luxury unavailable for scarce but complex SVs. However, the second stage, spacing-dependent filtering, creates the critical bottleneck distinguishing SV development from SNP array design. InDels exemplify this paradox: despite moderate probe design success (25.2%), their extreme genomic density necessitated a 1900-bp spacing threshold (versus 200-bp for SNPs), resulting in the lowest overall yield (0.09%) among all variant classes. This reflects biological inevitability: small indels cluster in hypervariable regions where probe spacing constraints force the sacrifice of 99.9% of candidates.

The contrasting efficiency profiles across SV classes (Table 1) illuminate design principles for future arrays. PAVs (9.3% overall yield) balance high probe success with moderate spacing constraints, offering the optimal compromise between development cost and functional relevance. Complex SVs (inversions 13.7%, translocations 18.4%, duplications 8.2%, CNVs 14.6%) show variable design success but 100% retention postdesign due to genomic scarcity, each successful complex SV represents high-information content that justifies inclusion regardless of breakpoint spacing. Notably, the 8.2% success rate for duplications reflects the particular challenge of capturing paralogous sequence variants without cross-hybridization.

Our empirical benchmarks establish realistic expectations: developing a 6K-SV array requires evaluating ~70 000 PAV candidates or ~ 7 million InDel candidates, a resource intensity that explains why SV chips remain rare in horticultural crops. For researchers initiating similar projects, we recommend prioritizing PAVs over small InDels when seeking large-effect regulatory variants [12], as the 0.09% InDel yield presents prohibitive cost barriers. Conversely, complex SVs should be prioritized despite low abundance; their 100% retention rate and high per-locus information content (often tagging gene regulatory changes undetectable by SNPs) offer exceptional functional return on design investment. The 40 K SNP-SV Chip’s composition, 34% complex SVs/PAVs versus 66% small variants, represents an empirically optimized balance for watermelon breeding, a ratio likely transferable to other crops with comparable genome complexity.

### Population genomics: refining the domestication understanding and germplasm classification

Our analyses corroborate the African single-origin model (Guo et al., 2019), providing fine-scale resolution that reveals complex speciation dynamics within the primary gene pool. The 40 K SNP-SV Chip data demonstrate that *C. mucosospermus*, though recognized as a distinct species based on seed mucilage and pericarp hardness, constitutes a genetically proximate sister species to West African wild *C. lanatus*, emerging as a differentiated lineage only at *K* ≥ 4 rather than constituting a distant outgroup. This pattern, when coupled with minimal genetic differentiation and shared genetic components (Pop2/3) with West African wild forms, reflects substantial genetic continuity likely maintained by historical or ongoing gene flow in sympatric West African populations, rather than deep phylogenetic divergence. This blurring of genetic boundaries between congeneric species aligns with observations in other crop wild relatives where primary gene pool members retain interfertility despite morphological specialization [26].

This finding operationally defines three germplasm pools for breeding: (i) the basal African–West Asian wild reservoir (including Pop4 lineages representing rare ancestral diversity); (ii) the specialized *C. mucosospermus* lineage (a distinct species genetically proximal to wild *C. lanatus* but characterized by unique seed mucilage traits) and (iii) the derived Asia–America-cultivated pool with minimal wild introgression. The strong geographic–taxonomic coupling (PC1: 68.2% variance) reflects the domestication bottleneck followed by rapid dispersal, while the delayed differentiation of *C. mucosospermus* (until *K* ≥ 4) suggests incomplete lineage sorting or introgression rather than strictly allopatric speciation. The detection of Pop4, a rare, highly differentiated component in <3% of accessions, echoes studies in rice and grape where high-density genotyping revealed hidden substructure within presumed uniform wild groups [27, 28]. This underscores that the African wild reservoir contains unexplored genetic strata, potentially harboring unique stress-resistance alleles.

### The power of structural variations in trait dissection: regulatory insights and the limits of rare-variant detection

The GWAS results validate the 40K SNP-SV Chip’s capacity to resolve the ‘hidden’ genetic architecture of morphological traits while exposing the statistical constraints inherent to its design.

#### Methodological justification for haplotype-based structural variation testing

We acknowledge that including flanking polymorphic sites (84 545 sites) rather than testing SVs as biallelic markers (7087 loci) increases the multiple testing burden. However, this approach is biologically necessary for SVs: unlike SNPs, SVs often influence phenotypes through *cis*-regulatory effects spanning multiple kilobases (e.g. the *LCYB* upstream PAV detected in this study). Treating each SV as a single point mutation would ignore the haplotypic context and potentially miss causal regulatory variants in imperfect LD with the breakpoint. The Bonferroni-corrected threshold (–log_10_(p) > 6.23) adequately controls family-wise error rate, while the absence of genomic inflation confirms that the observed associations (e.g. *LCYB* locus at *P* = 10^−21^) reflect true biological signals rather than LD-induced artifacts. Future iterations could employ the optimal Sequence Kernel Association Test (SKAT‑O) or burden tests to aggregate rare SV effects within genes, potentially improving power for polygenic traits. Applied to the current dataset, this haplotype-based approach successfully resolved known loci while revealing novel regulatory variants.

#### Flesh color provides a paradigmatic example of structural variation–enhanced resolution

The SNP/InDel data merely tagged a downstream haplotype block near *LCYB*, replicating known associations [29]. In contrast, the SV haplotype blocks analysis pinpointed a cluster of significant variants, including a PAV ~100 kb upstream and an inversion ~300 kb downstream of the coding sequence, distinct from the two nonsynonymous SNPs within *LCYB* previously reported in bi-parental mapping populations [22]. This discrepancy likely reflects ascertainment bias inherent to the chip design: probe selection prioritized low-frequency structural variants (MAF > 0.35 in the discovery panel enriched for wild diversity), whereas the previously characterized functional SNPs may be fixed or at high frequency in cultivated germplasm. Importantly, rather than viewing this as a limitation, our discovery of candidate distant regulatory SVs suggests a possible genetic architecture: lycopene accumulation may potentially be modulated by *cis*-regulatory structural variants. This emphasizes the biological significance of the slower LD decay observed in noncoding regions (Fig. 4C), where regulatory SVs can maintain long-range associations with target genes. The substantially stronger association signal of the upstream PAV (−log₁₀p = 20.76) compared to any coding-region SNP supports its potential functional significance, consistent with the major impacts of SV on gene expression regulation and fruit trait development in tomato [30] and transposon-mediated regulatory evolution in pepper [31].

#### Rind pattern: uncovering cryptic structural complexity

Similarly, while SNPs confirmed the major WDR gene locus on Chr6, the SV analysis unveiled the underlying complexity, 56 PAVs within the interval, and discovered entirely novel loci. The association of a methyltransferase gene on Chr10 with rind patterning implicates potential epigenetic regulation, a layer of control rarely captured by standard GWASs. Furthermore, significant SVs on Chr9 co-located with large-scale cryptic rearrangements (translocations, inversions) undetectable by short-read SNP calling. These findings align with evidence in apples and grapes, where SVs directly control key agronomic traits [32, 33].

#### Quantitative traits: power limitations and ascertainment bias

Conversely, comprehensive scans for yield-related traits (fruit weight, dimensions, SSC) yielded no significant associations. We attribute this primarily to limited statistical power: GWASs of polygenic traits typically require very large sample sizes to yield reproducible results and identify small-effect QTL [34], whereas our panel of 228 accessions was assembled for diversity representation rather than association mapping. The continuous, right-skewed distribution of fruit weight (skewness = 1.45, kurtosis = 4.27; Fig. S5) indicates phenotypic variation within a narrow range of cultivated forms, lacking extreme segregation expected for major QTL. The absence of inflation in QQ plots (λ = 0.84; Fig. S6) confirms that these null results reflect the mismatch between the chip’s allele frequency spectrum and trait genetic architecture, rather than analytical error. An additional compounding factor is the chip’s ascertainment bias toward wild diversity, a design feature advantageous for mining rare regulatory variants but suboptimal for yield optimization in elite pools.

### From ascertainment bias to breeding precision: leveraging rare-variant enrichment for wild introgression

The extreme left-skewed site frequency spectrum of the 40K SNP-SV Chip, i.e. 67.0% of SNPs/InDels and 74.3% of SVs exhibiting MAF < 0.05 (Fig. 4A), reflects a fundamental property of its design architecture rather than a technical flaw. This distribution arises from the mismatch between the discovery panel (28 accessions enriched for wild diversity) and the genotyping population (228 accessions, largely improved cultivars). Alleles present at intermediate frequencies in the discovery set became ‘rare’ (MAF < 0.05) in the improved cultivar population, while the higher proportion of rare SVs compared to SNPs/InDels aligns with the lower mutation rates and stronger purifying selection typically acting on SVs. Consequently, while this ascertainment bias limits power for detecting common QTL (MAF > 0.1) underlying domestication traits, it confers exceptional advantages for wild-to-crop introgression breeding.

#### Dual-generation deployment strategy

We propose a phased breeding strategy that transforms this ascertainment bias from a limitation into a diagnostic advantage. In cultivated germplasm, wild-specific SVs are rare (MAF < 0.05), making them difficult to track with conventional SNP arrays. However, in interspecific hybrid populations (F₁ or BC₁), these same alleles transition to intermediate frequencies (MAF ~0.25–0.50), precisely the range where the 40K SNP-SV Chip has maximal discriminatory power. The extended LD decay of SV markers (maintaining *r*^2^ > 0.1 over 10–70 kb compared to 10–20 kb for SNPs; Fig. 4B) further ensures robust tracking of large donor segments even with sparse marker coverage. (i) ‘Early-generation’ (F₁–BC₁) foreground selection employs the 7087 SV markers to track introgressed segments carrying resistance QTL from wild relatives (*C. colocynthis*, *C. amarus*) or seed-quality traits from *C. mucosospermus*. This stage exploits the frequency transition of wild-specific SVs to intermediate levels, enabling efficient monitoring of donor genome segments. (ii) ‘Advanced backcross’ (BC₂–BC₃) background selection utilizes the combined SNP-SV panel (*n* = 18 470 loci) to eliminate linkage drag and characterize recombination breakpoints with high precision, accelerating recovery of the elite cultivar genome while retaining targeted wild introgressions. This approach leverages the chip’s rare variant enrichment as a specialized tool for wild allele mining rather than a universal solution for all breeding stages. Therefore, the chip is highly recommended for tracking wild introgressions in early-generation breeding populations (F₁–BC₃).

#### Limitations for elite population improvement

Conversely, for improvement of polygenic domestication traits such as yield and SSC, statistical power remains the primary constraint: our 228-accession panel is underpowered to detect small-effect QTLs, and selective sweeps have further fixed beneficial alleles at these loci in modern cultivars, rendering them effectively monomorphic. Compounding this sample size limitation, the same ascertainment bias creates a ‘blind spot’ for common QTLs (MAF > 0.1). Consequently, the chip is not recommended for direct use in elite breeding pools for polygenic domestication traits (e.g. yield, SSC). Its utility in such contexts is limited unless (i) increasing population size with enhanced genetic diversity, (ii) supplementing with additional common-SNP markers to capture fixed alleles, or (iii) deploying in early-generation populations where allelic diversity is higher.

#### Future perspectives

While the current chip design prioritizes rare variant discovery, addressing the power limitations discussed above requires a dual-pronged strategy. First, for immediate utility in elite breeding pools, researchers may supplement the 40K SNP-SV Chip with additional common-SNP markers (MAF > 0.1) and expand population size to ensure adequate statistical power for polygenic trait optimization. Second, to enhance SV detection across the entire genus, expanding the probe set to include pangenomes from deeply divergent wild species (e.g. *C. naudiananus*) could create a universal tool with improved call rates in secondary and tertiary gene pools. Third, for early-generation introgression populations where our chip exhibits maximal discriminatory power, SV-assisted genomic selection should be prioritized to precisely track wild alleles while rapidly recovering elite genetic backgrounds. Ultimately, the 40K SNP-SV Chip establishes a foundation for SV-informed crop improvement, enabling a strategic shift from SNP-centric breeding toward comprehensive genomic diversity utilization.

## Materials and methods

### Single variant identification and development of the Watermelon 40K SNP-SV Chip

Motivated by the comprehensive structural-variation map generated in our recently assembled watermelon pan-genome [2], we set out to translate these SV data into a practical genotyping tool and therefore initiated the development of a watermelon SV array. Briefly, 27 representative accessions (13 cultivated and 14 wild) were *de novo* assembled with PacBio HiFi and Hi-C data and aligned to the G42 reference genome with minimap2. SVs ≥ 20 bp were simultaneously extracted from the SyRI comparison and cross-validated with long-read callers (sniffles, cuteSV, and SVIM), merged with SURVIVOR and genotyped across all assemblies.

Then the variants were selected as primary variant pool for further filtration using the parameters: Na <0.2, Het <0.15, and MAF >0.35. These primary variants were subjected to the same two-step filtering workflow previously established for SNP array construction (Guo et al., 2019). First, biological filters were applied: copy-number polymorphisms were retained only if detected in ≥2 accessions and supported by ≥5 split reads; InDels (20–50 bp) and larger SVs (> 50 bp) were required to exhibit *r*^2^ < 0.2 with adjacent SNPs to avoid signal redundancy. Second, probe-design parameters identical to those used for SNPs were adopted: (i) probe length 110 bp, (ii) GC content 30%–70%, (iii) no simple-sequence repeats or assembly gaps, and (iv) ≤5 genomic hits after BLAST against the G42 genome. After extraction, 6 851 083 high-confidence SV loci remained across all 27 accessions, including 6 777 958 insertions/deletions, 66 501 PAVs, and 6624 CNV/Duplications/Inversions/Translocations. From this pan-genome catalogue, 13 367 evenly distributed variants (6280 InDels, 6154 PAVs, and 933 others), together with 5114 core SNP markers, were selected for probe synthesis. Probe design employed a multiplexed capture strategy, utilizing the 40K platform’s 90 000-probe capacity: SNP loci were targeted by dual probes (one per allele); InDels and complex SVs used flanking probes (two per breakpoint) plus spanning probes where feasible (for variants <50 bp), with 2× redundancy to ensure capture efficiency. In total, 86 380 oligonucleotide probes (96% capacity, averaging ~4.7 per locus) were synthesized to constitute the final watermelon liquid-chip SV array (Table S1).

### Plant materials and phenotyping

Two successive populations were employed to evaluate the Watermelon 40 K SNP-SV Chip. (i) Initial test panel—12 accessions covering all seven *Citrullus* species (*C. naudinianus* PI 641945, *C. rehmii* PI 670011, *C. ecirrhosus* PI 674447, *C. colocynthis* PI 537300, *C. amarus* PI 482276, *C. mucosospermus* PI 595203, and *C. lanatus* landrace PI 254622, plus five cultivated *C. lanatus* cultivars, Sugarlee, JinSeLuoHuang, CC-1 M, G22, and the root-mutant line Mut426), employed to assess call rate, reproducibility and initial performance. (ii) Natural population—228 diverse accessions (7 *C. mucosospermus* and 221 *C. lanatus*) were subjected to the genotyping with 40K SNP-SV Chip. Detailed accession information and genotyping performance are provided in Table S3. While all 228 were included in downstream population genomic analyses (PCA, Admixture, GWAS), quality control identified eight accessions with call rates lower than 85% (primarily wild species), which were flagged but retained to avoid ascertainment bias toward cultivated germplasm.

The 228-accession panel was grown in a randomized complete-block design with three replicates at two locations: Xiashan Research Station, Institute of Advanced Agricultural Sciences, Peking University, Weifang, China (spring 2025), and the Experimental Station of Wuwei Li’an Seed Co., Ltd., Gansu, China (spring 2025). For each accession, five uniformly ripe fruits were harvested at 35 days after pollination (DAP). Fruit weight (FW, g), maximum transverse diameter (TD, mm), and longitudinal diameter (LD, mm) were recorded within 2 h of harvest. Total soluble solids (TSS, °Brix) were measured on flesh taken from the fruit center using a digital pocket refractometer (PAL-1; ATAGO). Rind stripe pattern was scored on a 1–6 scale (1 = serrated, 2 = netted, 3 = striped, 4 = radial, 5 = speckled, 6 = no markings). Flesh color was recorded on an ordinal scale from 0 to 5 representing progressive pigmentation intensity (0 = white, 1 = pale yellow, 2 = yellow, 3 = orange, 4 = pink, 5 = red). Both flesh color and rind stripe pattern were analyzed as quantitative traits using EMMAX, treating the ordinal scores as continuous variables for exploratory association mapping.

### Library preparation, probe hybridization, and sequencing

Genomic DNA was isolated from 10 pooled young-leaf disks per accession using a magnetic-bead kit (MolBreeding), quantified with Qubit 2.0 and checked for integrity on agarose gel. DNA was randomly sheared to 200–400 bp by ultrasonication, end-repaired, A-tailed, and ligated to barcoded adapters. AMPure XP beads were used to size-select 200–300 bp fragments, and the indexed library was amplified for eight cycles. Five-hundred nanograms of the finished library were dried with Block I/II, resuspended in 2 × Hyb buffer plus 60 ng/μl probes, and hybridized at 65°C for 2 h. Probe-bound targets were captured with DNA Probe Beads at 65°C for 45 min, washed serially with warm and room-temperature buffers, and amplified for 15 cycles (GenoBaits GBTS protocol; Guo et al., 2019). The final library was purified with AMPure XP beads, quantified with a Qubit 2.0 and verified to be 300–400 bp by gel electrophoresis, and sequenced on the BGI DNBSEQ-T7 platform.

### *In silico* analysis of sequence data

Paired-end reads were first cleaned with fastp v0.20.0 (−n 10 − q 20 − u 40) to remove adapters and discard any read pair containing >10 Ns or >40% bases with Phred quality ≤20. Clean reads were aligned to the watermelon reference genome with BWA-MEM (default settings). Variants were called following the GATK Best-Practice pipeline (HaplotypeCaller → GVCF → joint genotyping). For each capture probe, genotypes were extracted and filtered as follows: (i) positions with depth < 5× were set to NA (missing); (ii) heterozygous calls required ≥4 reads for each allele, otherwise the genotype was also set to NA. Small indels (≤40 bp) were genotyped using GATK HaplotypeCaller with indel-specific parameter optimization.

#### Single-variant genotyping and quality control

For all SV types, we required ≥5 supporting reads and applied uniform genotype thresholds: homozygous reference (<0.3 junction read proportion), heterozygous (0.3–0.7), and homozygous alternative (≥0.7); ambiguous signals (0.1–0.3 or 0.7–0.9) were conservatively flagged as missing. Specifically, PAVs and CNVs were identified by junction-spanning reads across breakpoints; inversions by reverse-forward (RF) orientation read pairs; and translocations by chimeric read pairs mapping to different chromosomes. To address uneven capture efficiency, we implemented: (i) depth normalization using core SNP median depth as scaling factor; (ii) probe-specific thresholds relative to flanking region depth (±1 kb); (iii) technical replicate concordance checks (≥98%); and (iv) manual integrative genomics viewer (IGV) inspection of GWAS-significant SVs.

### Genome-wide association study

Genome-wide association analyses were performed using the mixed linear model (MLM) implemented in EMMAX [35] to account for population structure and cryptic relatedness. Three distinct marker datasets were analyzed independently: (i) total (*n* = 6701), (ii) SNPs and InDels (*n* = 5681), and (iii) SV haplotype blocks (*n* = 84 545 polymorphic sites across 7087 SV loci). The SV dataset employed a haplotype-block approach to maximize statistical power for large-effect SVs. For each SV locus, we defined a physical interval spanning the breakpoints ±5 kb flanking regions and extracted all high-quality polymorphic sites (SNPs and small indels) within this window (*n* = 84 545 total sites across 7087 SV intervals, averaging ~12 sites per SV). This approach treats each SV as a haplotype background rather than a single point mutation, capturing potential causal regulatory variants in LD with the SV breakpoints. To control for the expanded testing burden, we applied a stringent Bonferroni correction (α = 0.05/84545 = 6.23). To control for population stratification, the first three principal components (PCs) from PCA were included as fixed-effect covariates, and a kinship matrix (*K*) calculated using simple matching coefficients was incorporated as random effects to account for relatedness among accessions. Flesh color (0–5) and rind stripe pattern (1–6) were treated as ordinal quantitative traits and analyzed as continuous variables. All quantitative traits (fruit weight, dimensions, °Brix, etc.) were fitted as continuous variables. Significance thresholds were determined using Bonferroni correction (α = 0.05): 5.13 for Total markers (0.05/6701), 5.06 for SNP/InDel only (0.05/5681), and 6.23 for SV only (0.05/84545). Manhattan plots and QQ plots were generated using CMplot and qqman packages in R. Genomic inflation factors (λ) were calculated to assess model fit. Manhattan plots and QQ plots were generated using ggplot2 and dplyr in R, with color schemes optimized using the viridis palette.

### Data analysis and visualization

Population structure analyses were performed using PLINK [36] for PCA calculation, Admixture (v1.4) for model-based clustering, and VCF2Dis [37] with FastME (v2.0; [38]) for phylogenetic tree construction. The resulting phylogenetic trees were annotated and visualized using Rstudio. Chromosomal distribution maps were generated using the Advanced Circos module in TBtools [39]. All other plots (PCA, Manhattan, QQ, LD decay) were generated using ggplot2 [40] and dplyr in R (v4.2.0), with color palettes optimized using the viridis package.

## Supplementary Material

Web_Material_uhag221

## Data Availability

The data underlying this article are available in the article and in its online supplementary material.
