# Supplementary material for: Development and application of a 40K liquid chip enriched with structural variation markers for watermelon breeding
Source: Hortic Res. 2026 Jun 3;13(11):uhag221. doi: 10.1093/hr/uhag221 (PMC13722873; doi:10.1093/hr/uhag221)
Supplement: Supplementary file 1 [file web_material_uhag221.zip › Supplementary figures.pdf]

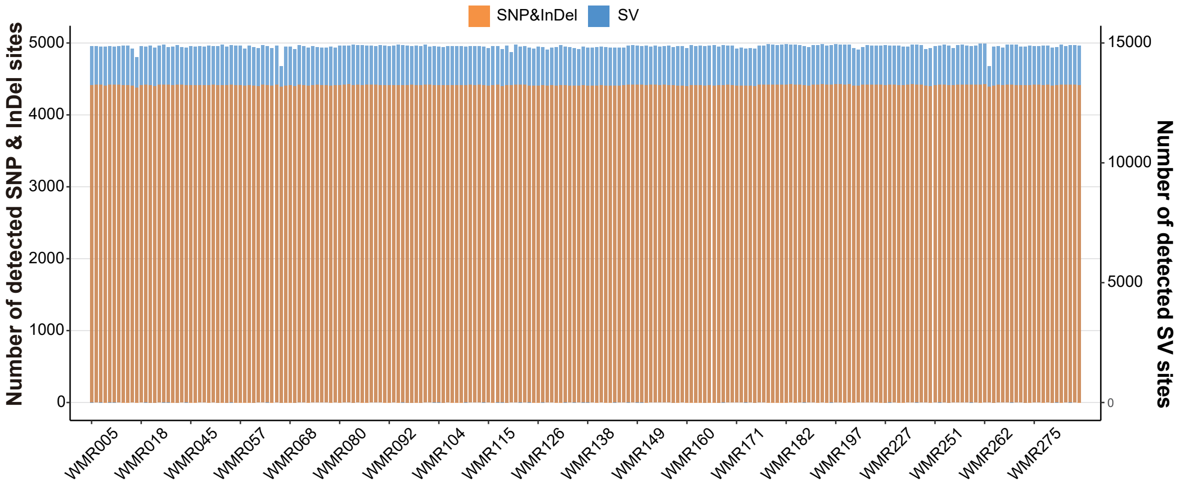

SNP&InDel
SV
Number of detected SNP & InDel sites
Number of detected SV sites
0
1000
2000
3000
4000
5000
0
5000
10000
15000
WMR005
WMR018
WMR045
WMR057
WMR068
WMR080
WMR092
WMR104
WMR115
WMR126
WMR138
WMR149
WMR160
WMR171
WMR182
WMR197
WMR227
WMR251
WMR262
WMR275

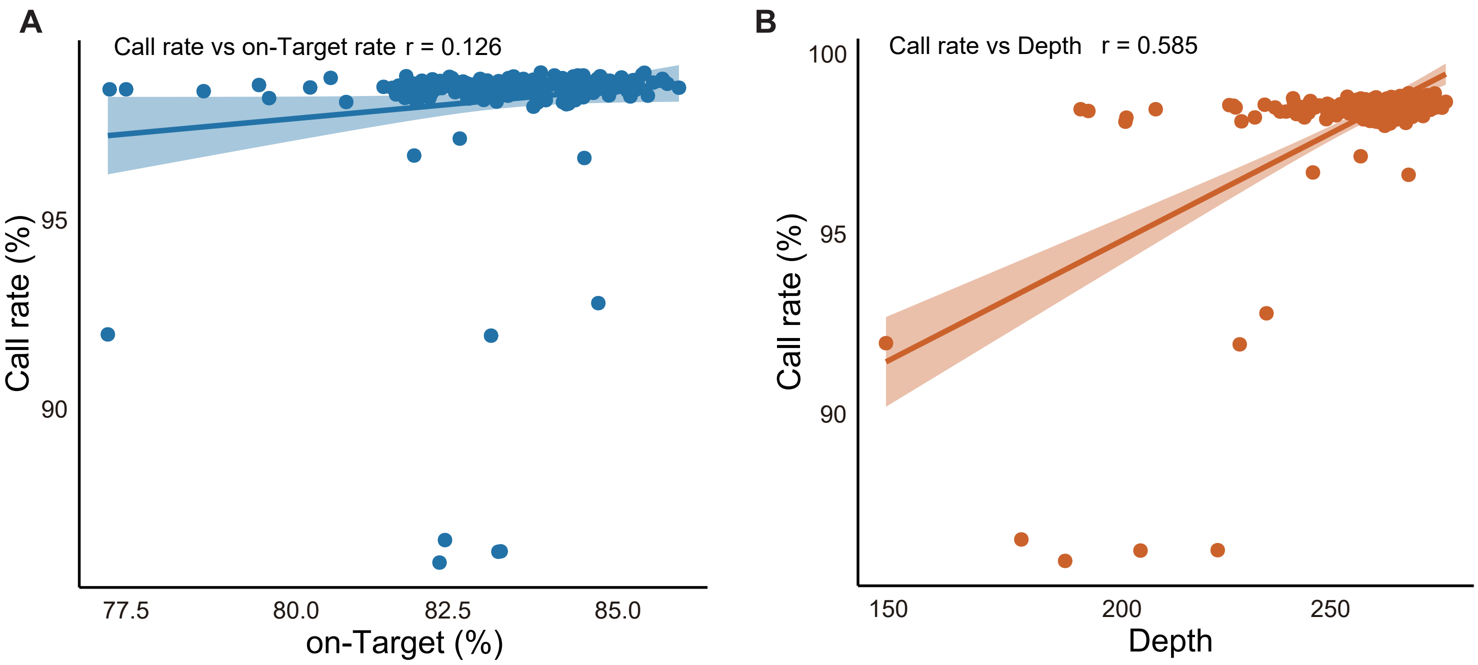

A
Call rate vs on-Target rate r = 0.126
Call rate (%)
95
90
77.5
80.0
82.5
85.0
on-Target (%)
B
Call rate vs Depth r = 0.585
100
95
90
Call rate (%)
150
200
250
Depth

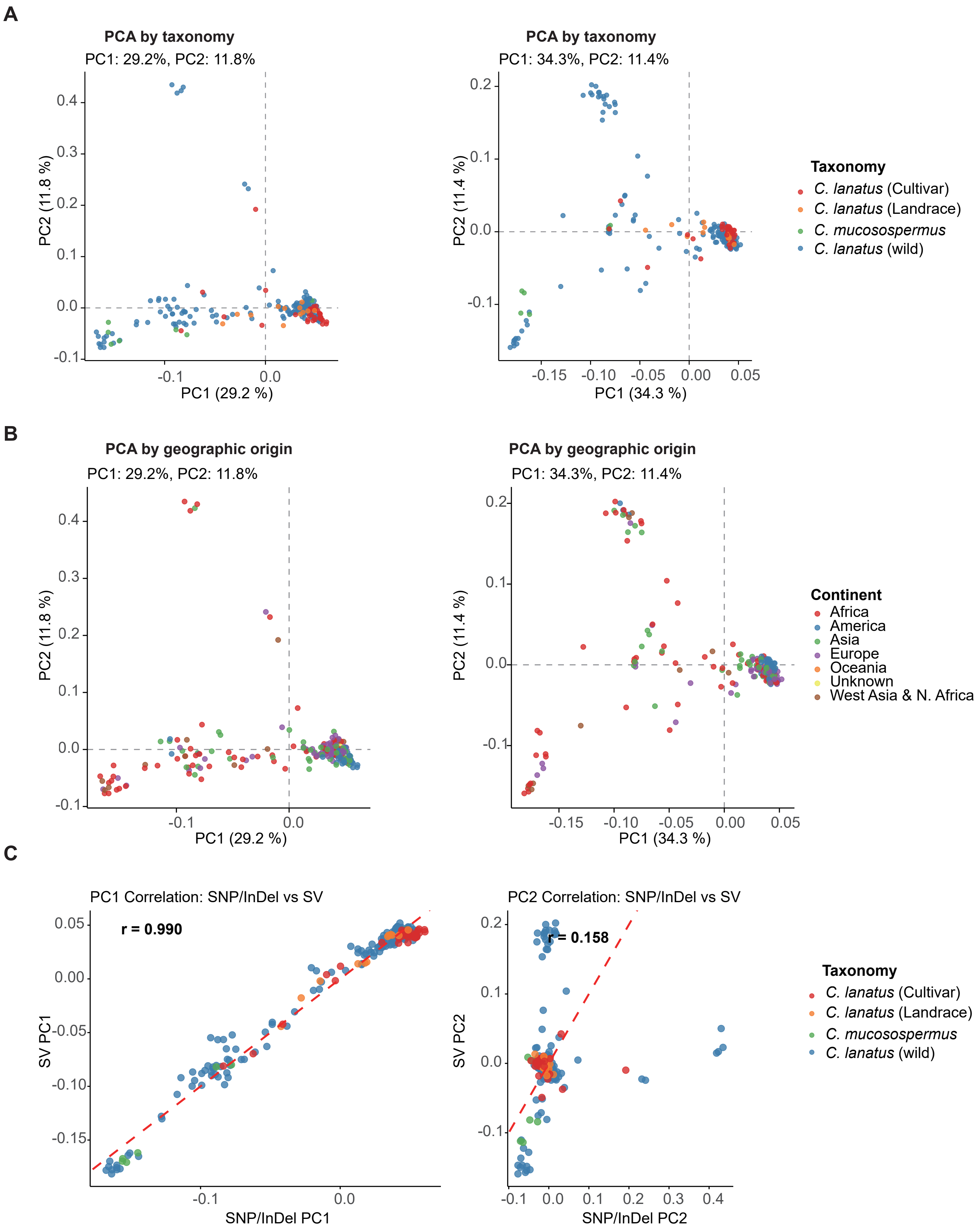

A
PCA by taxonomy
PC1: 29.2%, PC2: 11.8%
PC2 (11.8 %)
PC1 (29.2 %)
PCA by taxonomy
PC1: 34.3%, PC2: 11.4%
PC2 (11.4 %)
PC1 (34.3 %)
Taxonomy
C. lanatus (Cultivar)
C. lanatus (Landrace)
C. mucosospermus
C. lanatus (wild)
B
PCA by geographic origin
PC1: 29.2%, PC2: 11.8%
PC2 (11.8 %)
PC1 (29.2 %)
PCA by geographic origin
PC1: 34.3%, PC2: 11.4%
PC2 (11.4 %)
PC1 (34.3 %)
Continent
Africa
America
Asia
Europe
Oceania
Unknown
West Asia & N. Africa
C
PC1 Correlation: SNP/InDel vs SV
r = 0.990
SV PC1
SNP/InDel PC1
PC2 Correlation: SNP/InDel vs SV
r = 0.158
SV PC2
SNP/InDel PC2
Taxonomy
C. lanatus (Cultivar)
C. lanatus (Landrace)
C. mucosospermus
C. lanatus (wild)

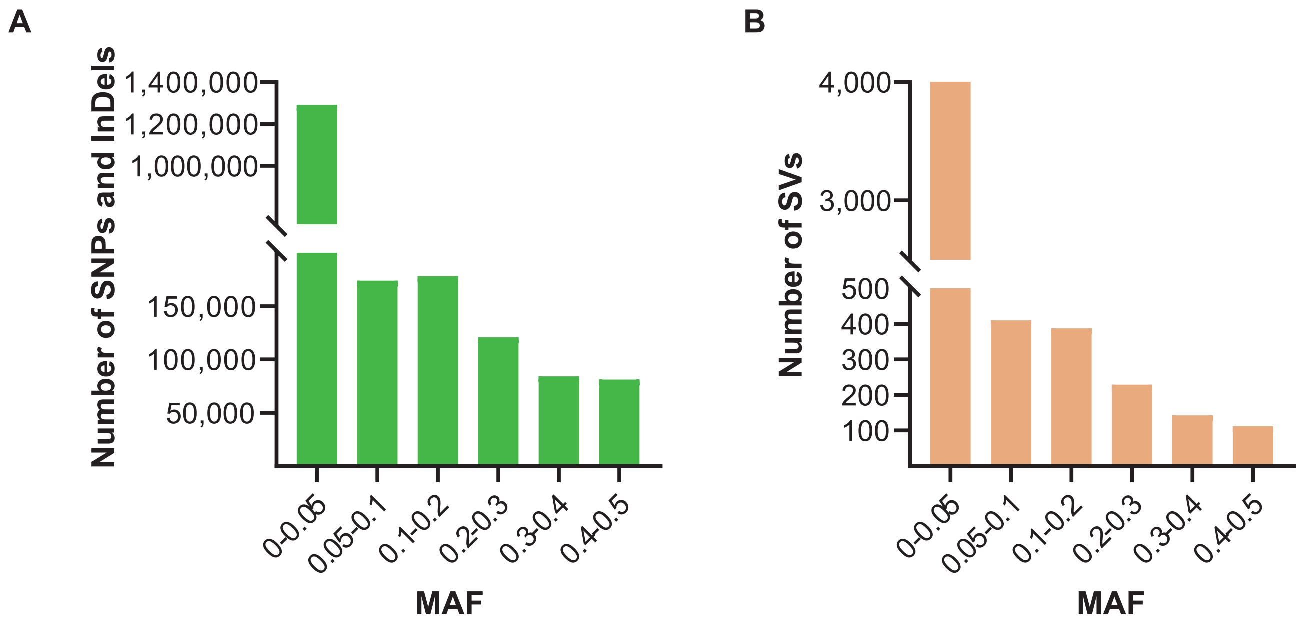

A
B
Number of SNPs and InDels
1,400,000
1,200,000
1,000,000
150,000
100,000
50,000
Number of SVs
4,000
3,000
500
400
300
200
100
0-0.05
0.05-0.1
0.1-0.2
0.2-0.3
0.3-0.4
0.4-0.5
MAF

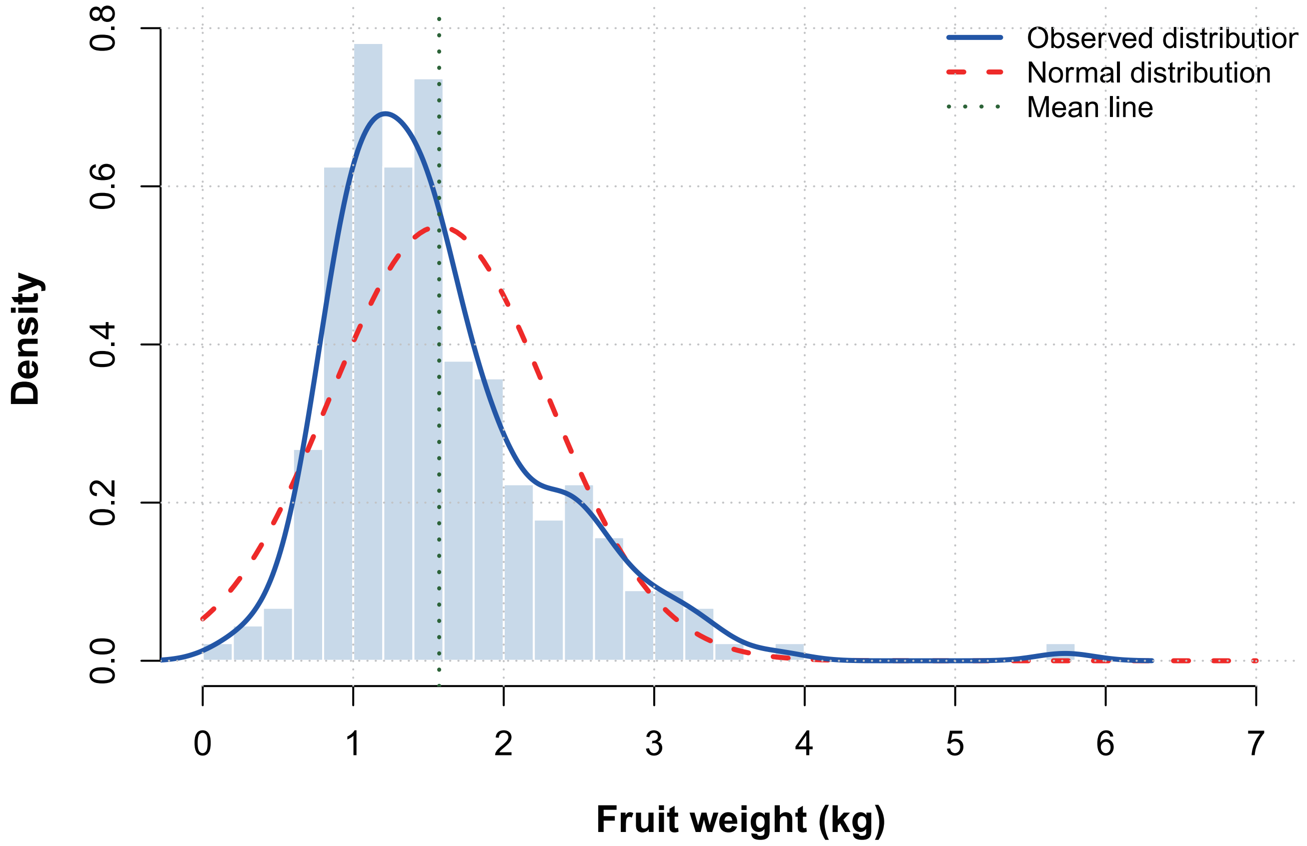

Density
0.0
0.2
0.4
0.6
0.8
0
1
2
3
4
5
6
7
Fruit weight (kg)
Observed distributior
Normal distribution
Mean line

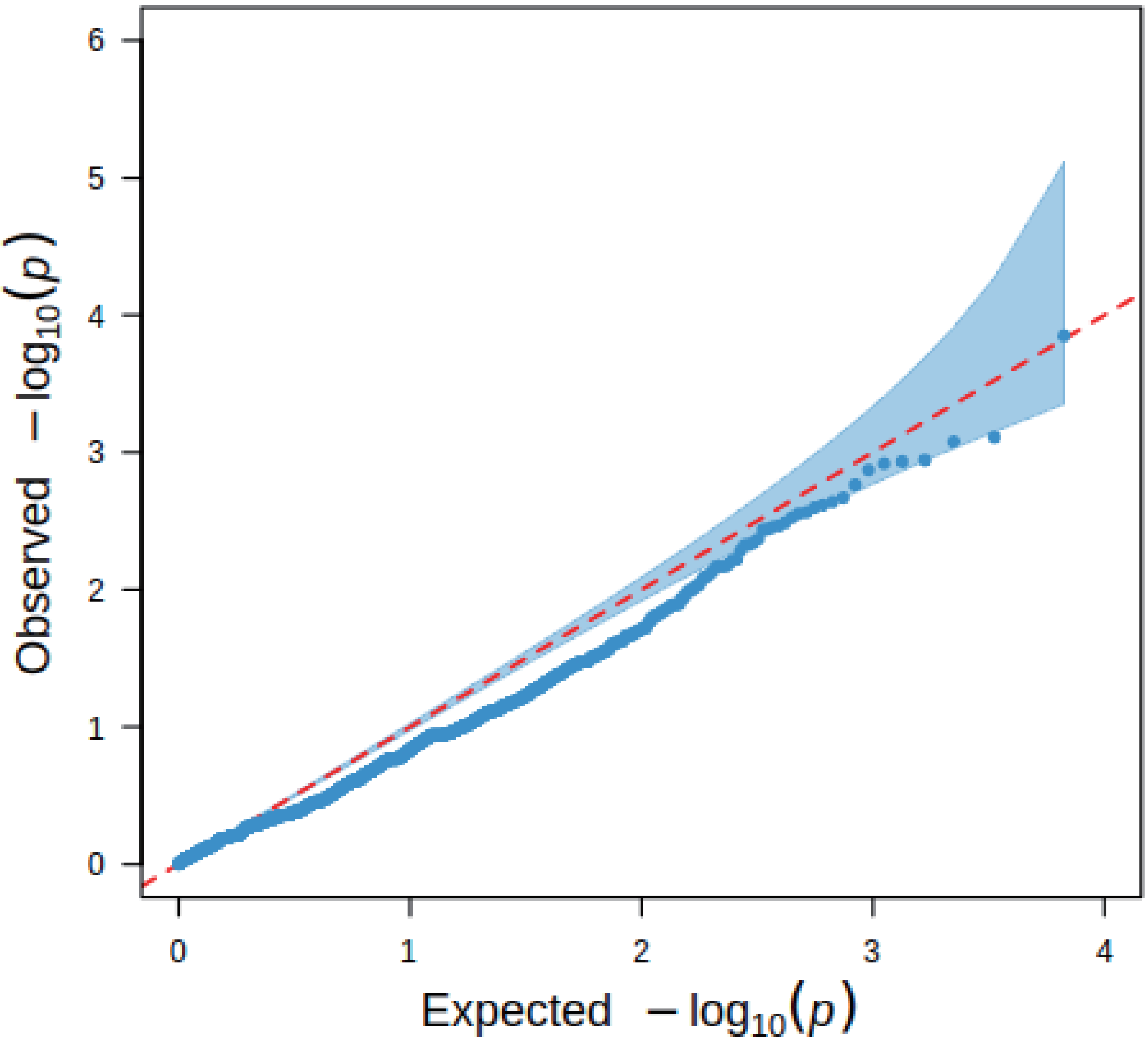

Observed $-\log_{10}(p)$
Expected $-\log_{10}(p)$
0
1
2
3
4
5
6
0
1
2
3
4
